# Artificial Neural Network (ANN) as a Tool to Reduce Human-Animal Interaction Improves Senegalese Sole Production

**DOI:** 10.3390/biom9120778

**Published:** 2019-11-25

**Authors:** Juan M. Martínez-Vázquez, David G. Valcarce, Marta F. Riesco, Vicent Sanz Marco, Morito Matsuoka, Vanesa Robles

**Affiliations:** 1 IEO, Spanish Institute of Oceanography, Planta de Cultivos El Bocal, Barrio Corbanera, 39012 Monte, Santander, Spain; juanma.martinez@ieo.es (J.M.M.-V.); dgvalcarce@gmail.com (D.G.V.); riesco.mf@gmail.com (M.F.R.); 2 Cybermedia Center, Osaka University 1-32 Machikaneyama, Toyonaka, Osaka 560-0043, Japan; v.sanzmarco@cmc.osaka-u.ac.jp (V.S.M.); matsuoka@cmc.osaka-u.ac.jp (M.M.); 3 Department of Molecular Biology, Universidad de León, 24071 León, Spain

**Keywords:** Senegalese sole, production science, biomass, artificial neural network

## Abstract

Manipulation is usually required for biomass calculation and food estimation for optimal fish growth in production facilities. However, the advances in computer-based systems have opened a new range of applied possibilities. In this study we used image analysis and a neural network algorithm that allowed us to successfully provide highly accurate biomass data. This developed system allowed us to compare the effects of reduced levels of human-animal interaction on the culture of adult Senegalese sole (*Solea senegalensis*) in terms of body weight gain. For this purpose, 30 adult fish were split into two homogeneous groups formed by three replicates (*n* = 5) each: a control group (CTRL), which was standard manipulated and an experimental group (EXP), which was maintained under a lower human-animal interaction culture using our system for biomass calculation. Visible implant elastomer was, for the first time, applied as tagging technology for tracking soles during the experiment (four months). The experimental group achieved a statistically significant weight gain (*p* < 0.0100) while CTRL animals did not report a statistical before-after weight increase. Individual body weight increment was lower (*p* < 0.0100) in standard-handled animals. In conclusion, our experimental approach provides evidence that our developed system for biomass calculation, which implies lower human-animal interaction, improves biomass gain in Senegalese sole individuals in a short period of time.

## 1. Introduction

The Senegalese sole (*Solea senegalensis*) has become one of the most promising flatfish species cultured in Europe, reaching 1600 tons in 2017 [1], which represents an increase of 266% of total production over the last four years. One of the main reasons for its rising is the expansion of facilities dedicated to the culture of this species and the optimization of the management protocols, allowing for an increment of biomass stocked. 

An adequate biomass control of the tanks is a critical factor to determine the food intake requirements and to ensure the maximum growth performance of the cultivated fishes [2]. The traditional estimation procedures of weighting subsamples from the fish population implies a stressful situation, causing adverse effects on the stock productivity and welfare of the animals, which could lead to disease development or death [3]. Over recent years, different mechanisms based on image analysis for quantifying the biomass have been tested in many commercial species [4], showing different levels of accuracy in their predictions [5]. However, most of these systems are designed for their application on offshore cages with pelagic species [6,7,8], and their use on flatfish land-based farms is scarce [9,10]. Moreover, the particular characteristics of each species should be carefully considered for developing adequate and accurate systems for biomass calculation. In the particular case of *Solea senegalensis*, overlapping of flatfish individuals in the bottom of the tank jeopardize the use of previously developed artificial recognition systems [9]. Tagging of the breeders for their individualized control is also a common procedure in aquaculture, generally via passive integrated transponder (PIT; [11]), although the use of visible implant elastomers (VIE) is becoming more prominent, mainly applied in fingerlings [12,13]. Both require the injection of an exogenous element, being a traumatic event for the fish, but afterwards the requirements of interaction are low, not affecting normal development [14]. 

We hypothesized that the development of a specific image recognition system using artificial neural networks for measure prediction in *Solea senegalensis* will provide an accurate biomass estimation, reducing human handling and improving animal weight gain. The use of this system would benefit the aquaculture industry, reducing the cost associated with manual labor and providing faster fish growth. In order to test this, in our study we analyzed the influence of standard periodic biomass samplings of adult cultured Senegalese sole against a low interaction methodology relying on a new image analysis predictor. Fish were tagged with VIE, allowing for the individualized growth monitoring of each fish and the checking of estimator accuracy.

## 2. Materials and Methods

### 2.1. Ethics Statement

The experimental design and all protocols and procedures, including animals of the present study, were approved by the institutional Animal Care and Use Committee (authorization number PI-10-16) at the Marine Culture Plant *El Bocal* of the Spanish Institute of Oceanography in Santander, Spain. All animals were standard manipulated according to the Guidelines of the European Union Council (2010/63/EU), following Spanish regulations (RD/2013) for the use of laboratory animals. 

### 2.2. Group Formation and Animal Management

Adult Senegalese soles (*n*=30) (unknown sex) born and reared in captivity at the Spanish Institute of Oceanography facilities were sampled at day 0 of the survey. The fish were anesthetized in a bath of 40 ppm of diluted clove oil (Guinama, Valencia, Spain) for two minutes, prior to being measured and weighed using an ICS429 balance (Mettler, Toledo, Spain). During this sampling, fish were also tagged with yellow visible implant elastomers (VIE; Northwest Marine Technology, Shaw Island, WA, USA) on their dorsal area, using an individualized code for each animal based on the number and position of marks. This system allowed us to identify them individually, avoiding withdrawing them from the tank. 

After this, fish were split into two groups with different levels of human interaction throughout the experiment. Detailed information about the type of human-animal interaction factors received by each group, as well as the kind of animal stimuli and frequency of factors are included in Supplementary Table S1. Each group was formed by three tank replicates of *n* = 5 animals. The soles were distributed to reach approximately the same initial biomass density of 1.67 ± 0.009 kg/m^2^ per tank in all replicates, trying to have similar size dispersion in all of them. In the control group (CTRL), the fish followed a standard management schedule that included monthly samplings of weight and size; meanwhile, in our experimental group (EXP), human interaction was reduced to the minimum possible, involving only the feeding and siphoning operations, and VIE visualization throughout the experiment.

Fish were maintained in 1390 L grey fiberglass tanks in open flow circuit, with a 1.7 m^3^/h water renovation rate and moderate aeration. They were placed inside an industrial warehouse set up with an artificial photoperiod of 16 h of light and 8 h of darkness. The light intensity was reduced using a black shading mesh over the tanks that allowed for a maximum light intensity at the water surface of 50 lux. The mesh only covered 3/4 parts of the total surface of the tank, leaving the remaining area as feeding and control window. The fish were subjected to a natural thermoperiod during the first 20 days of experimentation, fluctuating from 11.10°C to 12.50 °C. Then, the temperature was modified to progressively increase until reaching 16 °C. A reproductive response was then induced by programming a temperature fluctuation from 16°C to 18°C, and decreased again to 16°C in cycles of three and four days, according to previous protocols [11,15] in order to facilitate sexing of the fish.

Animals were fed daily with a commercial diet of Europa 5^®^ pellets from Skretting España S.A, Burgos, Spain, to an adjusted quantity of the 0.5% biomass of each tank.

### 2.3. Manual and Visual Samplings

The total duration of the study was 126 days. The fish from the control group (CTRL) were withdrawn from their tanks once per month, and their biometrical parameters were measured manually for monitoring growth, following standard protocols in animal production centers. Animals included in the experimental replicates (EXP) were not manipulated, and biomass was calculated using the image analysis developed. A final weight and size sampling were performed in all six tanks at day 126, determining the sex of each fish during the process by external examination of abdominal swelling. In both groups, before final manual sampling, a top-down photograph of each tank was taken with an Olympus Stylus Tough-6020 digital camera (Olympus Corporation, Tokio, Japan) at a height of 1.5 m over the water’s surface. Each fish in the image was identified by the visualization of their VIE tags with an ultraviolet (UV) flashlight.

### 2.4. Biomass Estimation by Image Analysis

The pictures obtained were digitally processed to provide an alternative sampling method that reduces human-animal interaction. In order to create a suitable artificial vision system for sole fish, two different convolutional neural networks (CNNs) were used: Inception_v4 and a novel CNN developed for biomass calculation. Both neural networks were trained using train-test split. Besides, a reference object inside the tanks was used to help the artificial vision system to correctly measure the fish in the photo. In our experiments, this reference object is a three-dimensional (3D) printed white hemisphere (diameter: 4.2680 cm). The reference was chosen due to it being easy to detect for the neural network; its measures are known, and in a top-view image, the reference object is always a circle. 

Supplementary Figure 1 shows the steps performed by the artificial vision system to obtain the biomass estimation. The artificial vision system has four different phases:

(1) In the first phase, the user sends a top-view tank photo to our server. The server receives the image and resizes it to obtain, at least, the height or the width in 1024 pixels. The aspect of the image is not modified, and all of the colors of the original picture are retained.
(2) In the second phase, we train the last layer of Inception_v4 to detect all fish in the tank. However, to train this CNN, we need to train it using sole fish images. To the best of our knowledge, there are no available datasets to detect sole fish. For this reason, we created a training dataset of 2000 sole fish images and 200 reference object images, contained in several fish tanks. This Inception_v4 model can identify the sole fish and the reference object, even if only a small part is visible, such as its head or tail. As a result, this CNN has 96% fish detection accuracy and 100% of reference object detection accuracy.
(3) In the third phase, after all fish and the reference object in the picture have been identified and framed, our artificial vision system calculates the real size of all fish. This system uses the reference object framed in the picture to calculate the distance between the camera and the object. It calculates how many pixels the reference object framed contains, and makes a relation between its size in the real world and its pixels in the image. So, the system makes its own calibration and then calculates the size of every fish in the image. Thus, the application can determine the fish measurements, height and width, with a measurement error chance of 0.5% and 0.4%, respectively.
(4) In the fourth phase, a novel CNN is created that is able to estimate the fish biomass using the height and the width of the fish. Supplementary Figure 1 shows the nine layer CNN developed for this purpose. This neural network has been trained with a dataset that contains the height, width, and biomass of 400 fish. Additionally, it has been used *adam* as an optimizer, together with a learning rate of 0.001 with a batch size of four.

Once the artificial vision system has finished, the server returns to the user an image with each framed fish and its identifying number attached. Moreover, an Excel file is sent to the user with the following information: the identifier number of each fish, together with its predicted height (cm), width (cm), and biomass (g). Currently, this artificial vision system is under invention patent protection (P201930392; under evaluation).

### 2.5. Data Analysis

Results are expressed as the mean ± standard error of mean (S.E.M.), except for individual growth rates. The comparison of biometrical data between groups at 0 and 126 days were analyzed using a t-Student for paired samples. Statistical analyses were performed with Prism8 (GraphPad Software, San Diego, CA, USA). *p*-values < 0.05 were considered statistically significant.

## 3. Results

### 3.1. Visible Implant Elastomer Tagging

**Figure 1:**
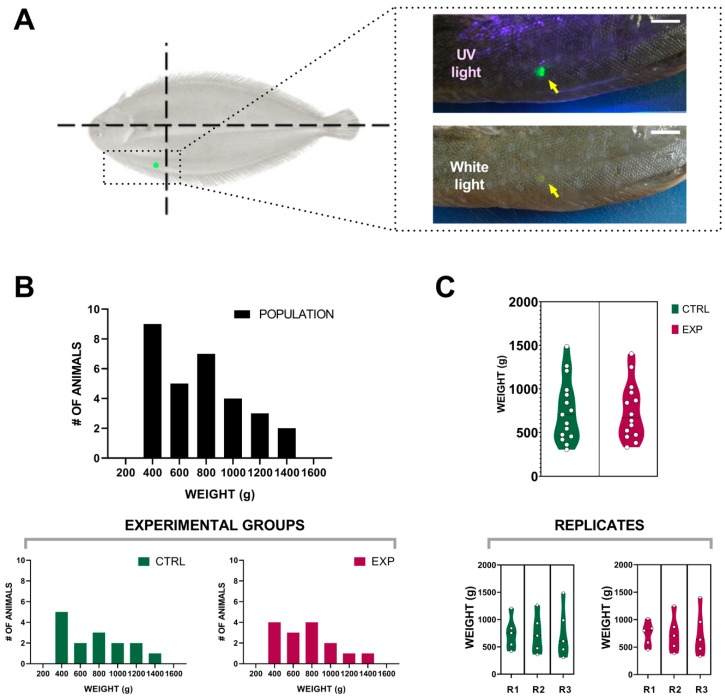
(**A**) *Solea senegalensis* visible implant elastomers (VIE) tagging. Schematic representation of the tagging area and photographs of a tag example under white and ultraviolet (UV) light. Scale: 1 cm. (**B**) Histograms showing the distribution of total population at day 0 and the resulting experimental groups. (**C**) Violin plots representing initial weight of all animals as well as replicate values included within control (CTRL) and experimental (EXP) groups.

In order to individually track each animal involved in the experiment, we used visible implant elastomers (VIE). To our knowledge, our study is the first to use this technology in *Solea senegalensis* individuals. The VIE injection was simple to perform in the anesthetized fish, obtaining a high retention tag that was easily monitored throughout the experiment (Figure 1A). The tag was easily readable through the water column (60 ± 5 cm) by using an ultraviolet light source (82 mW). No superficial wounds were recorded in any of the animals within the experiment. Our results validate the use of this system for this species.

### 3.2. Effect of the Intensity Human-Animal Interaction on Sole Biomass Gain

**Figure 2:**
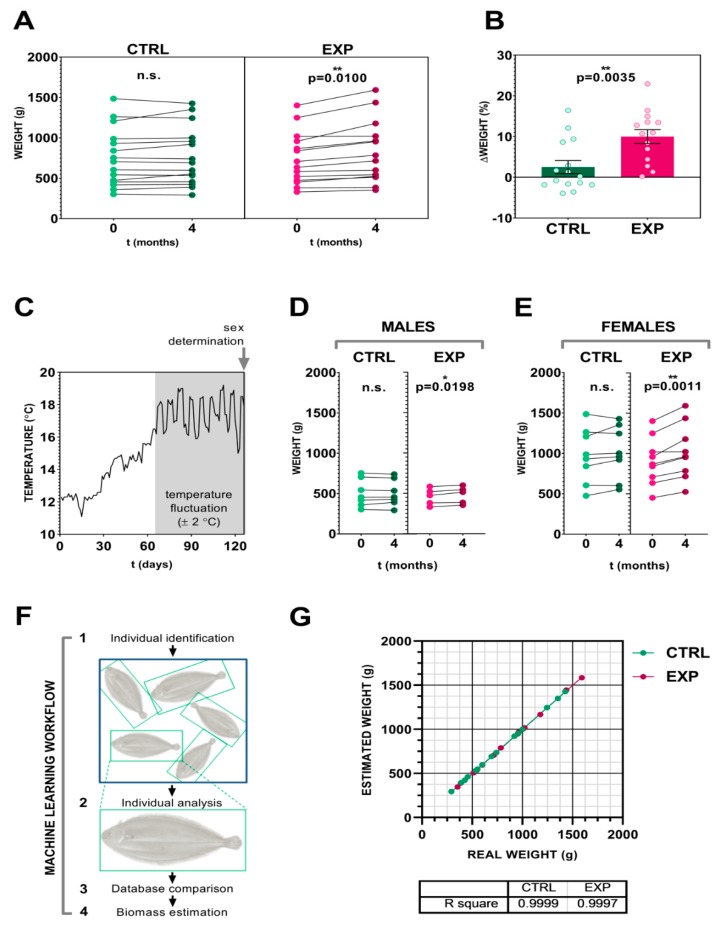
(**A**) Before-after graph showing *Solea senegalensis* weight at time 0 and 4 months. (**B**) Bar graph comparing individual body weight increment of control (CTRL) and experimental (EXP) groups. (**C**) Temperature profile registered during the experiment. Grey area indicates the reproductive induction period. (**D**) Analysis of body weight in males and (**E**) females individuals. (**F**) Machine learning workflow. (**G**) Dot plot showing estimated weight reported by image analysis vs real weight. Asterisk shows statistically significant differences.

The total population included in this experiment was split into two homogeneous groups. Special care was taken in order to avoid considerable variations in the initial population distribution in terms of body weight (Figure 1B). The replicated tanks (three per experimental condition) were also similar in terms of initial total biomass: 1.6637 ± 0.0138 kg/m^2^ for CNTRL tanks and 1.6742 ± 0.0134 kg/m^2^ for EXP tanks (Figure 1C). Only one death was reported throughout the trial in one of the EXP tanks, two days after the initial tagging. The consequent necropsy showed no signs of illness, and the death was ascribed to stress derived from initial sampling. After four months of experimental conditions (Supplemental Table S1), the animals included in the control group (standard manipulated) did not report statistically significant differences in body weight (0.7561 ± 0.0926 kg at *t* = 0 vs. 0.7730 ± 0.0935 kg at *t* = 4 months). On the other hand, those animals under lower human-animal interaction culture conditions reported a statistically significant difference (*p* = 0.0100) between the initial and final sampling (0.7449 ± 0.0873 kg vs. 0.8267 ± 0.1024 kg) (Figure 2A). Furthermore, all ofthe individuals in the EXP group (100%) reported a body weight increment, contrary to CTRL group where only 8/15 showed an increase (53.33%).

Results regarding individual body weight increment are included in Figure 2B. After four months of low-interaction culture, animals within this group reported statistically significant (*p* = 0.0035) higher values (weight increment (Δ): 9.9810 ± 1.712 kg) when compared to standard-handled animals (Δ: 2.4990 ± 1.6010 kg).

The programmed temperature fluctuation (Figure 2C) successfully allowed performing a sex determination of the animals included within the experiment. The purpose of this sex description was to analyze data avoiding sex bias. Obtained results (Figure 2D,E) confirmed the overall conclusions. Only statistically significant body weight increases were registered in experimental cultured conditions in both males (*p* = 0.0198) and females (*p* = 0.0011) individuals.

### 3.3. Image Analysis and Artificial Neural Networks as a Tool for Precise Fish Biomass Prediction

Considering the reported evidence of the human interaction effect on biomass variable, it seems necessary to find an alternative to routine samplings involving the direct manipulation. In this study we used an image analysis approach to overcome an excess of animal stimulation. Figure 2,F shows the machine learning workflow used in the present study. The results provided by the algorithm are highly accurate, as can be observed in Figure 2,G. The estimated weight against real weight plot reported a R^2^= 0.9999 for CTRL and a R^2^= 0.9997 for EXP. These results strongly support the use of this approach on sole weight sampling.

## 4. Discussion

Worldwide flatfish (Pleuronectiformes) aquaculture is increasing steadily. *Solea senegalensis* belongs to the group of the most cultured species of this order, together with *Paralichthys olivaceus*, *Scophthalmus maximus*, *Hippoglossus hippoglossus*, *Platichthys flesus,* and *Solea solea* [16]. This species has high economic importance in South-western European countries, and as a consequence, during the last years the scientific community has focused on providing new insights in many different areas of knowledge, from molecular biology [17,18,19] to immunology [20,21] or development [22]. 

The zootechnics in aquaculture are in constant evolution, in order to reach the maximum production rates, making the initial economical investment the more profitable as possible in the shortest time. This is leading to the development of farming systems able to maintain the environmental conditions at very stable levels, like the recirculating aquaculture systems (RAS). 

In the same way, the incorporation of new technologies for stock management directed to increase both production and animal welfare is a necessary step forward in the aquaculture. As a canon, aquaculture farmers perform manual or machine assisted samplings from a subset of the stock to obtain mean weight data. Human-interaction under captivity conditions as part of animal environment is a key factor that can be linked to fish growth, reproduction, immune systems, and fitness of the animals [23]. It is generally accepted that most domestication processes involve behavioral, morphological, and physiological changes.

The main objectives of this work were the following: 1) to develop a customized computer-based system for weight prediction that could provide an accurate biomass calculation reducing manual handling of animals, and 2) to study the effects of reduced human interaction on Senegalese sole growth. To assess our goals, we worked with adult individuals under two different culture conditions in terms of human-derived stimuli (routine protocol (CTRL) against reduced level protocol (EXP)) (Supplementary Table S1). After a short term (four months) of culture conditions, our results revealed a clear weight gain in those animals subjected to less interaction with humans in a general way (Figure 2A,B), as well as taking sex into account (Figure 2E,F). These results were not reported in the routine-sampled animals, indicating an inverse correlation between biomass increment and human-derived stimulation in this flatfish species. The individual comparison was able thanks to the implementation of VIE as tagging protocol. Until now, no publications have been reported in literature using this type of tagging in *Solea senegalensis*. Our experiment has corroborated the suitability of these molecules for animal tracking. This technology may be used for routine protocols instead of PIT for the management of a small population. VIE tagging is less invasive, effortless to apply, easy to monitor without withdrawing the animals, and cheaper than PIT protocols.

Our results inferred the need to develop a biomass calculation system able to reduce fish-human interaction. Machine learning (computer systems that automatically improve their performance through experience [24]) has become a major worldwide focus of attention during recent years, together with big data technologies and high-performance computing. This, together with emerging fields like deep learning or big data managing [25], allows for performing countless new applied approaches in the aquaculture field. Moreover, animal welfare is a crucial factor to take into account in culture protocols, and new technological advances must be at the service of this purpose. In line with this, there already exist examples of the use of machine learning in aquaculture [26,27]. Under this framework, we have applied an innovative protocol, particularly adapted to this species, based on image recognition and machine learning (artificial neural networks) for biomass prediction. The used algorithm has been successfully accurate in its purpose (Figure 2,G and H) without the necessity of extra installations in the facility. 

## 5. Conclusions

The use of this system in industrial or semi-industrial aquaculture facilities could improve economic efficiency by reducing handwork routines needed for biomass monitoring tasks. Moreover, the accurate prediction of biomass, used for food supply calculation, and the reduced fish-human interactionproduced clear beneficial effects on animal growth in only four months of culture. These results allow us to envision that this developed system may positively impact Senegalese sole farming.

## Supplementary Materials

The following are available online at https://www.mdpi.com/2218-273X/9/12/778/s1, Table S1: Description of the human-animal interaction factor, the stimuli received by the fish and detailed frequency and number of events of each factor. Figure S1: A. Phases of the artificial vision system developed to estimate the biomass of fish by image analysis. B. CNN architecture used to predict the biomass of a fish.
