# Supplementary material for: Artificial Neural Network (ANN) as a Tool to Reduce Human-Animal Interaction Improves Senegalese Sole Production
Source: Biomolecules. 2019 Nov 25;9(12):778. doi: 10.3390/biom9120778 (PMC6995621; doi:10.3390/biom9120778)

Appendix A

Supplementary Materials: Table S1. Description of the human-animal interaction factor, the stimuli received by the fish and detailed frequency and number of events of each factor. Figure S1. A Phases of the artificial vision system developed to estimate the biomass of fish by image analysis. B.CNN architecture used to predict the biomass of a fish.

Table S1. Main cultured fish disturbances

Human-animal interaction factor	Stimuli from humans	Tanks: CTRL Frequency	Tanks: CTRL Cumulative	Tanks: EXP Frequency	Tanks: EXP Cumulative
Fedding	visual, olfactory, gustatory and auditory	Daily	126	Daily	126
Partial emptying of the drain	auditory	Daily	126	Daily	126
Water temperature measurement	visual and auditory	Daily	126	Daily	126
Profilactic treatment (Peroxide)	visual, olfactory, gustatory and auditory	Fortnight	8	Fortnight	8
Manual biometric measurement	tactil, visual, olfactory, gustatory and auditory	Monthly	4	-	0

Figure S1. Convolutional neuronal network workflow

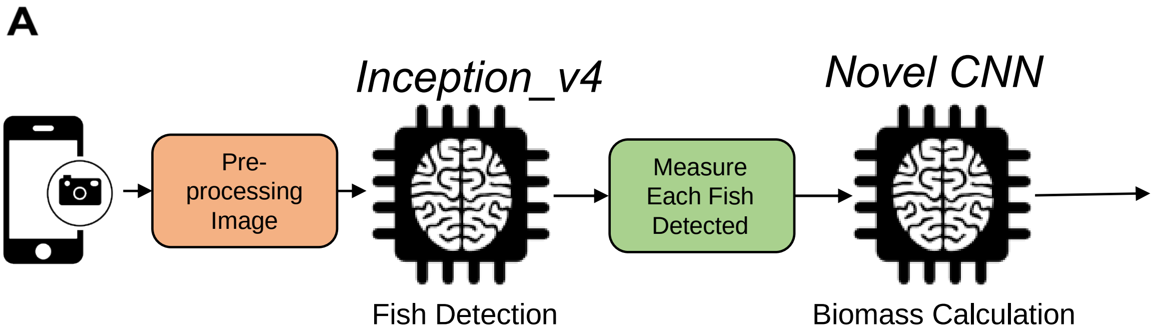

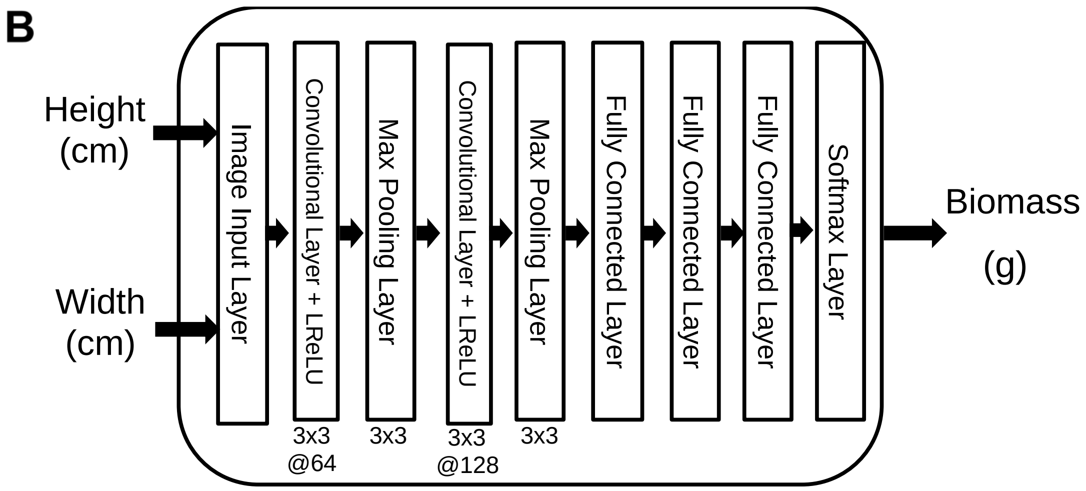

Supplement: Supplementary file 1 [file biomolecules-09-00778-s001.pdf]
